# PGC-1α as a Pivotal Factor in Lipid and Metabolic Regulation

**DOI:** 10.3390/ijms19113447

**Published:** 2018-11-02

**Authors:** Ching-Feng Cheng, Hui-Chen Ku, Heng Lin

**Affiliations:** 1 Department of Pediatrics, Taipei Tzu Chi Hospital, Buddhist Tzu Chi Medical Foundation, New Taipei City 23142, Taiwan; chengcf@mail.tcu.edu.tw (C.-F.C.); ku311@hotmail.com (H.-C.K.); 2 Institute of Biomedical Sciences, Academia Sinica, Taipei 11529, Taiwan; 3 Department of Pediatrics, Tzu Chi University, Hualien 97004, Taiwan; 4 Institute of Pharmacology, Taipei Medical University, 250 Wu-Hsing Street, Taipei 11031, Taiwan; 5 Department of Physiology, School of Medicine, College of Medicine, Taipei Medical University, Taipei 11031, Taiwan

**Keywords:** PGC-1α, metabolic homeostasis, adipose tissue, mitochondria

## Abstract

Traditionally, peroxisome proliferator-activated receptor γ coactivator 1α (PGC-1α), a 91 kDa transcription factor, regulates lipid metabolism and long-chain fatty acid oxidation by upregulating the expression of several genes of the tricarboxylic acid cycle and the mitochondrial fatty acid oxidation pathway. In addition, PGC-1α regulates the expression of mitochondrial genes to control mitochondria DNA replication and cellular oxidative metabolism. Recently, new insights showed that several myokines such as irisin and myostatin are epigenetically regulated by PGC-1α in skeletal muscles, thereby modulating systemic energy balance, with marked expansion of mitochondrial volume density and oxidative capacity in healthy or diseased myocardia. In addition, in our studies evaluating whether PGC-1α overexpression in epicardial adipose tissue can act as a paracrine organ to improve or repair cardiac function, we found that overexpression of hepatic PGC-1α increased hepatic fatty acid oxidation and decreased triacylglycerol storage and secretion in vivo and in vitro. In this review, we discuss recent studies showing that PGC-1α may regulate mitochondrial fusion–fission homeostasis and affect the renal function in acute or chronic kidney injury. Furthermore, PGC-1α is an emerging protein with a biphasic role in cancer, acting both as a tumor suppressor and a tumor promoter and thus representing a new and unresolved topic for cancer biology studies. In summary, this review paper demonstrates that PGC-1α plays a central role in coordinating the gene expression of key components of mitochondrial biogenesis and as a critical metabolic regulator in many vital organs, including white and brown adipose tissue, skeletal muscle, heart, liver, and kidney.

## 1. Introduction

The peroxisome proliferator-activated receptor gamma coactivator-1 (PGC-1) family includes ligands of multiple nuclear or non-nuclear receptors that control the expression of specific genes regulating cell metabolism. The first discovered member of the PGC-1 family, a 91 kDa nuclear protein [1] identified in brown adipose tissue (BAT) in mouse studies of cold-induced thermogenesis, was called peroxisome proliferator-activated receptor gamma (PPARγ) coactivator 1α (PGC-1α) [2]. The biological activity of PGC-1α is tightly controlled at several levels: by transcriptional control (of multiple promoter regions), alternative splicing of transcripts, and post-translational modification (e.g., phosphorylation, acetylation, or methylation). This activity results in several mRNA isoforms—PGC-1α-a, PGC-1α-b, PGC-1α-c, and NTPGC-1α—that enable cellular adaptation to various environmental conditions [3]. Studies have shown that PGC-1α can be used in different tissues with different coactivators to induce changes in lipid oxidation, energy homeostasis, mitochondrial mass, and insulin sensitivity. Here, we review these studies.

## 2. PGC-1α Can Regulate Lipid Metabolism

As a transcription factor, PGC-1α can bind to targets such as PPARα, PPARβ/δ, and PPARγ, which coordinate the expression of mitochondrial genes and indirectly contribute to fatty acid (FA) transport and utilization [4]. Furthermore, PGC-1α upregulates the expression of several genes of the tricarboxylic acid cycle [5] and the mitochondrial FA oxidation pathway [6]. PGC-1α also regulates the expression of nuclear and mitochondrial genes that encode components of the electron transport system and oxidative phosphorylation (OXPHOS) via nuclear respiratory factors 1 and 2 (NRF-1 and -2) and estrogen-related receptor α (ERRα) coactivation. These effects can increase the expression of mitochondrial transcription factor A (mtTFA), which is known to control mtDNA replication and transcription and therefore regulate cellular oxidative metabolism [7]. Accordingly, the augmented expression of cytochrome c, cytochrome-c-oxidase subunits II and IV, and adenosine triphosphate (ATP) synthase also result from PGC-1α activation [8,9,10,11].

Another noteworthy effect of PGC-1α is its ability to stimulate peroxisomal activity such as the oxidation of long-chain and very-long-chain FAs [12]. Briefly, PGC-1α level is positively correlated with the ability of cells to fully oxidize FA, an effect that may reduce intramuscular lipid deposition and improve tissue insulin sensitivity. Chromatin immunoprecipitation assays have shown that the mechanism of this effect includes the coactivation of liver X receptor α (LXRα), which stimulates PGC-1α binding to the LXR response element in the FAS promoter. In addition, muscle-specific PGC-1α expression in MPGC-1α transgenic mice exacerbated de novo free fatty acid (FFA) synthesis as well as FA esterification and triacylglycerol (TAG) accumulation [13]. Furthermore, PGC-1α is involved in lipid distribution and may upregulate FAT/CD36, FABPpm, and FATP1 mRNA and protein expression in mitochondrial fractions. The latter effect was confirmed solely in murine FAT/CD36 and FABP3 cells [14]. 

## 3. PGC-1α as a Coactivator for Metabolic Homeostasis in Skeletal Muscle

Muscle adjusts to endurance exercise by promoting mitochondrial biogenesis, angiogenesis, and changes of fiber composition [15,16,17]. Chinsomboona et al., had reported that mice lacking PGC-1α in skeletal muscle failed to increase capillary density in response to exercise. This study showed that β-adrenergic stimulation of a PGC-1 α/estrogen-related receptor alpha (ERRα)/vascular endothelial growth factor (VEGF) axis modulates exercise-induced angiogenesis in skeletal muscle [18] and truncated PGC-1α can lead to hypoxic induction of VEGF and angiogenesis in skeletal muscle [19]. In addition, PGC-1α activates transcription in cooperation with myocyte enhancer factor-2 (Mef2) and acts as a target for calcineurin signaling, which has been involved in slow fiber gene expression [20]. Skeletal muscle-specific PGC-1α knock-out mice demonstrate a shift from oxidative type I and IIa toward type IIx and IIb glycolytic muscle fibers [21]. Rasbach et al., reported that PGC-1α–mediated switch to slow, oxidative fibers in vitro is dependent on hypoxia-inducible factor 2 α (HIF2α), and mice lacking HIF2α in muscle increase the expression of genes and proteins related to a fast-twitch-fiber-type switch [22]. Transgenic mice with mildly elevated muscle levels of PGC1α are also resistant to age-related obesity and diabetes and show a prolonged lifespan [23]. These results strongly suggest that PGC1α expression in skeletal muscles can significantly contribute to regulating systemic energy balance. Recent studies also demonstrated that muscle contraction may induce the secretion of molecules called myokines, which enables the crosstalk between skeletal muscle and other organs such as adipose tissue, bone, liver, kidney, and brain; in this sense, skeletal muscle can be considered an endocrine organ. Indeed, several myokines discovered in the past decade via secretome analysis include interleukin-6, irisin/fibronectin type III domain-containing protein 5 (FNDC5), myostatin, interleukin-15, brain-derived neurotrophic factor (BDNF), β-aminoisobutyric acid, meteorin-like, leukemia inhibitory factor, and secreted protein acidic and rich in cysteine (SPARC).

**Figure 1:**
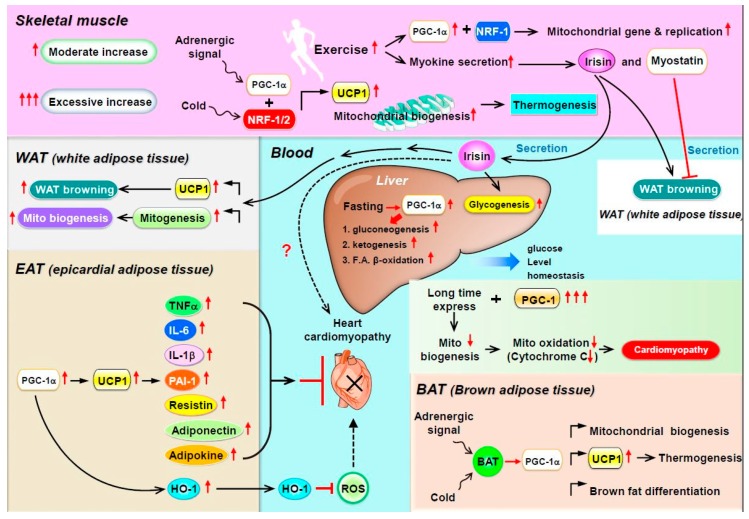
Schematic description of peroxisome proliferator-activated receptor gamma coactivator-1α (PGC-1α) function in various organs. Traditionally, in brown adipocytes (BAT) and white adipocytes (WAT), mitochondrial biogenesis and BAT gene expression are regulated by PGC-1α. Adrenergic stimulation and lower temperature trigger signaling cascades, including the upregulation of UCP-1 level, thereby resulting in body thermogenesis. In skeletal muscle and WAT, the transcriptional activity of PGC-1α is responsible for the expression of gene networks that control glucose uptake, glycolysis, fatty acid (FA) oxidation, tricarboxylic acid cycle, oxidative phosphorylation (OXPHOS), mitochondrial biogenesis, and protein uncoupling. Therefore, increasing exercise will increase mitochondrial gene biogenesis and secretion of myokines (such as irisin), which results in WAT browning and liver gluconeogenesis to prevent obesity and insulin resistance. In epicardial adipose tissue (EAT), increased heme oxygenase 1 (HO-1) expression depends on the PGC-1α–UCP-1 axis activity, which then decreases free radicals and reactive oxygen species (ROS) production, thus reducing cardiomyopathy. However, whether increased expression of cytokines such as TNF-α, IL-6, or adipokines by the PGC-1α–UCP-1 axis can reduce cardiomyopathy or not is still unclear.

Several myokines are regulated by PGC-1: irisin/FNDC5, myostatin, and *BDNF* [24]. (1) Irisin is a PGC-1α-dependent myokine. In mice with muscle-specific PGC-1α overexpression, PGC-1α induces the expression of a membrane protein, FNDC5, and exercise triggers the cleavage of FNDC5 to generate irisin and then secreted into the bloodstream, which elevates energy expenditure in subcutaneous adipose tissue via adipocyte browning [25]. This process implies that PGC-1α overexpression with exercise may increase the expression of uncoupling protein 1 (UCP-1) and eventually increase the browning of white fat cells [25]. Recently, mass spectrometry was used to measure circulating irisin levels in humans in an antibody-independent manner; irisin levels were increased by both short and prolonged period exercise [26,27]. Under physiological conditions, irisin stimulates glucose uptake and lipid metabolism via the activation of AMP-activated protein kinase (AMPK) [28,29,30] and is also involved in muscle growth by inducing insulin-like growth factor 1 and suppressing myostatin [31]. In addition to having effects on muscle, exogenous administration of irisin induces adipocyte browning in subcutaneous fat in mice via p38 mitogen-activated protein kinase (MAPK) and extracellular signal-regulated kinase 1/2 (ERK1/2) [32]. In the murine liver, irisin stimulates glycogenesis but reduces gluconeogenesis and lipogenesis by regulating GSK3, FOXO1, and SREBP2 [33,34,35]. (2) Myostatin is an autocrine and paracrine hormone secreted by muscle fibers and the only myokine with inhibited secretion during muscle contraction and exercise [36]. In addition to its local involvement in muscle atrophy [37], myostatin can also modulate metabolic homeostasis by regulating adipose tissue function [38,39,40]. The inhibition of myostatin was found to ameliorate the development of obesity and insulin resistance in mice fed a high-fat diet, presumably by mechanisms promoting lipolysis and mitochondrial lipid oxidation in adipose tissue and liver [41]. In addition, Dong et al., showed that inhibition of myostatin resulted in the conversion of white adipose tissue (WAT) to brown adipose tissue (BAT), while enhancing fatty acid oxidation and increasing energy expenditure. Inhibition of myostatin increased PGC-1α expression and irisin production in muscle. Irisin stimulated browning via mediating muscle-to-fat cross talk [42]. Myostatin knockout mice are characterized by increased expression and phosphorylation of AMPK in muscle, which subsequently activates PGC1α and Fndc5. This study demonstrated that Fndc5 is upregulated and secreted from muscle to induce browning of WAT in myostatin knockout mice [43]. (3) BDNF is known primarily as a molecule released by the hypothalamus and as a key element regulating neuronal development, plasticity, and energy homeostasis [44]. Cao et al., found that hypothalamic overexpression of BDNF via recombinant adeno-associated virus (rAAV) duplicated the enriched environment (EE)-associated activation of the brown fat program and lean phenotype. This study suggested that induction of hypothalamic BDNF expression in response to environmental stimuli results in selective sympathoneural regulation of white fat browning and increased energy dissipation [45]. Wrann et al., showed hippocampal BDNF gene expression [46]. PGC-1α knockout mice show decreased FNDC5 expression in the brain. Overexpression of FNDC5 increases BDNF expression in primary cortical neurons. Furthermore, peripheral delivery of FNDC5 to the liver leads to elevated blood irisin and increased BDNF expression in the hippocampus. Taken together, this study links endurance exercise and the significant metabolic mediators, PGC-1α and FNDC5, with BDNF expression in the brain [46] (Figure 1).

## 4. PGC-1α as a Coactivator in WAT Browning, Thermogenesis, and Mitochondrial Biogenesis

Much of the adaptive thermogenesis in small mammals takes place in BAT. BAT is morphologically and metabolically different from WAT and partly exerts opposite physiological functions. Adipocytes from BAT contain multiple small triglyceride-filled droplets as well as a large number of mitochondria. In addition, their mitochondria contain a specific UCP-1, expressed only in brown adipocytes. Genetic studies with mice lacking UCP-1 or PGC-1α in adipocytes indicated that (1) PGC-1α is the only protein that can powerfully activate the UCP-1 enhancer in non-BAT cell lines and (2) when pharmacologically introduced into white adipocytes, PGC-1α induces mitochondrial gene expression and mitochondrial biogenesis. Finally, PGC-1α is a downstream target of adaptive thermogenesis in BAT via adrenergic receptor activation [47,48], the key mechanism in brown-fat differentiation in in vitro cell cultures and in vivo cellular responses to cold exposure [24]. Brown fat and skeletal muscle, in which PGC-1α is highly expressed and can be induced by cold or adrenergic stimuli with enhanced mitochondrial biogenesis, are the two main contributing tissues in adaptive thermogenesis via the adrenergic receptor PGC-1α–UCP-1 axis. Scarpulla and collaborators [49,50] identified and cloned two novel transcription factors, NRF-1 and -2, that bind to the promoter region of the mitochondrial genes β-ATP synthase, cytochrome-c, cytochrome-c-oxidase subunit IV, and mtTFA. PGC-1α has a major effect on the NRF system. When introduced into muscle cells in vitro, PGC-1α greatly induces the gene expression of NRF-1, NRF-2, and mtTFA. Furthermore, PGC-1α interacts directly with NRF-1 and co-activates its transcriptional activity [51] (Figure 1).

## 5. PGC-1 Controls Cardiac Energy Metabolism in Healthy or Diseased Myocardia

In mammalian embryos, proliferating cardiomyocyte precursor cells rely on glycolysis as their major energy source, and mitochondrial tissue and oxidative metabolism are poorly developed. Once precursor cells differentiate into mature cardiomyocytes, a shift occurs from glycolysis to FA metabolism as the main provider of the entry point for mitochondrial oxidative phosphorylation, which in mature heart cells yields most of the energy [52]. Therefore, during neonatal development, the healthy myocardium increases its rate of β-oxidation while simultaneously decreasing glycolytic activity. Eventually, adult heart muscle derives ~90% of its energy from oxidative phosphorylation in mitochondria, which occupy only ~30% of cardiomyocyte volume [53,54]. During various cardiac disease processes, such as hypertrophy or ischemia-induced cardiomyopathy, both the inhibition of mediators of mitochondrial oxidative phosphorylation (cytochrome-c-oxidase subunits) and the expression or activity of metabolic enzymes involved in oxidative phosphorylation [55,56] were noted [57]. These processes of cardiac remodeling result in a gradual decrease in mitochondrial biogenesis [58], and ATP is utilized for maintaining ion homeostasis rather than for force production during cardiomyocyte contraction; this process leads to irreversible hypertrophy or dilated cardiomyopathy. However, during prolonged periods of cardiac remodeling, cardiomyocyte energy metabolism is regulated by the actions of various transcription factors and their coactivators, such as the PGC-1 family. PGC-1α has been shown to interact with three families of transcription factors: (1) the PPAR family, which regulates the expression of genes involved in FA oxidation; (2) the ERR family; (3) NRF-1 [2,59,60,61], which controls genes that are involved in mitochondrial oxidative phosphorylation and the electron transport chain [62,63]. In cardiomyocytes, PGC-1α is considered a master regulator of metabolism because it co-activates PPARs, ERRs, and NRFs [4,64] and may thereby control the entire metabolic phenotype of cardiomyocytes [7]. 

In the heart, the interrelationship between PGC-1α and PPARα plays an important role in regulation of the expression of enzymes involved in FAO and uptake pathways [65] and may be involved in regulation of mitochondrial biogenesis [66]. PGC-1α loss of function in murine heart exhibited a damage to mitochondrial respiratory function and reduced expression of genes involved in several mitochondrial metabolic pathways [67,68,69]. Hearts from PGC-1α KO mice showed reductions in mitochondrial enzymatic activities and ATP levels [67]. Arany et al., had shown that PGC-1α KO mice are prone to develop of heart failure in response to transverse aortic constriction (TAC). Furthermore, induction of PGC-1α in cells via catecholamine treatment can reverse the mitochondrial genes inhibition, suggesting that PGC-1α may be a potential therapeutic target in heart failure [68]. In addition, PGC-1α deficient mice cause energy metabolic derangements in multiple systems [69]. Conversely, overexpression of PGC-1α in adult mice had shown a moderate mitochondrial proliferation, abnormal mitochondrial architecture and severe cardiac dysfunction [70], and constitutive overexpression of PGC-1α in murine heart resulted in unconstrained mitochondrial proliferation in cardiac myocytes leading to a dilated cardiomyopathy [10]. 

Cardiac energy substrate metabolism is disturbed in the hypertrophic and failing heart. The myocardium switches from dependence on fatty acid oxidation (FAO) to glucose utilization in the failing heart, mainly anaerobic glycolysis [71,72,73,74]. These alterations in energy substrate preference are regulated, at least partially, by the downregulation of the genes involved in OXPHOS and FAO and the PPARα–PGC-1α complex [56,73,75,76,77,78]. The expression levels of PPARα and PGC-1α are reduced in several mice models of pressure overload, hypertensive heart disease [68,75,79], ischemic heart disease [57,80,81,82], hypoxia [76], and genetically engineered mouse models of heart failure [83,84,85]. Additionally, under pathologic conditions, PPARα activity is inhibited by the lower levels of the heterodimeric partner, retinoid X receptor (RXR) [86] and by direct phosphorylation, dependent on the extracellular signal-related kinase and mitogen-activated protein kinase (ERK–MAPK) pathway [75]. These findings suggest that deactivation of the cardiac PPARα–PGC-1α axis is an important component of the switch in energy metabolism in the failing heart. It remains to be addressed whether the deactivation of the oxidative metabolism and the PPARα–PGC-1α complex in the hypertrophied and ischemic heart is adaptive or maladaptive. The increment of myocardial reliance on anaerobic glycolytic pathways for ATP production is likely an adaptive response to reduce oxygen consumption. Indeed, partial inhibitors of FAO exhibited a promising therapeutic effect for cardiac disease [87,88,89]. Liao et al., had reported that overexpression of the GLUT1 glucose transporter can prevent pressure overload-induced heart failure [90]. Moreover, overexpression of PGC-1α [83] and PPAR agonists [91,92,93] can prevent cardiac hypertrophy or improve cardiac myocyte contractility. 

Cardiovascular disease is extraordinarily widespread in diabetic patients. Cardiomyopathy in diabetic subjects that occurs in the absence of known risk factors (hypertension, hyperlipidemia, etc.) is often referred to as “diabetic cardiomyopathy” [94,95,96,97]. Many studies have proposed that abnormalities in myocardial energy metabolism play an important role in the pathogenesis of diabetic cardiomyopathy. Indeed, the diabetic heart relies nearly exclusively on mitochondrial FAO for ATP requirements [98,99,100,101]. The expression levels of PPARα, PGC-1 α, and various target genes involved in FAO are increased in the murine insulin-resistant [66] and diabetic heart [102,103,104]. Moreover, transgenic mice that overexpress PPARα exclusively in the heart (MHC-PPARα mice) demonstrate a cardiac metabolic phenotype similar to that observed in the diabetic heart, including accelerated rates of FAO, reduction in glucose uptake and utilization, and repression of the mitochondrial biogenic response [66,102]. Mitochondria isolated from diabetic rodents showed reduced rates of OXPHOS [105,106] and decreased efficiency in ATP synthesis [107,108], likely due to increased uncoupled respiration [108]. The importance of PPARs and PGC-1α in the modulation of cardiac energy metabolism makes these regulatory pathways attractive therapeutic targets for diabetic cardiomyopathy.

In summary, increased PPARα and PGC-1α expression with the marked expansion of mitochondrial volume density and oxidative capacity accompany normal cardiac growth during postnatal maturation. Conversely, pathologic hypertrophy is associated with decreased PPARα–PGC-1α expression and/or activity and diminished reliance on oxidative mitochondrial metabolism, which leads to intramyocardial cell lipid accumulation. Finally, gain-of-function studies with PGC-1α overexpression in mice revealed that the extent of cardiomyopathy is primarily determined by the amount of PGC-1α that could be detected in the heart and, more importantly, the moment and duration of its emergence. Thus, both the synthesis and the moment of appearance of PGC-1α play important roles in the regulation of myocardial metabolism and mitochondrial biology (Figure 1).

## 6. Is PGC-1 a Paracrine Regulator in Epicardial Adipose Tissue?

Recently, a new type of adipose tissue, epicardial adipose tissue (EAT), was found in the heart of patients undergoing open-heart surgery. EAT is physically located next to the myocardium within the lateral wall of the right ventricle and the anterior wall of the left ventricle and surrounds the right coronary and left-anterior descending coronary arteries [109]. Similar to WAT, EAT shows high rates of lipogenesis but also high degrees of WAT lipolysis and thus serves as a local triacylglycerol (TAG) store [110]. EAT contains about five times more UCP-1 mRNA than WAT and also shows high expression of many genes of beige adipose tissue [111], that is, CD137, PRDM16, PGC-1α, C/EBPβ, and PPARα. The present understanding of the potential physiological roles of EAT includes: (1) the release of free FAs as energy to the myocardium under conditions associated with high metabolic demands, (2) the expression of the thermogenic protein UCP-1 in response to cold exposure, and (3) the expression and secretion of specific molecules for cardiovascular protection by vasocrine and paracrine pathways. EAT contributes to cardiovascular protection and vessel remodeling by secreting various paracrine factors. Several EAT-derived factors or cytokines, such as tumor necrosis factor alpha (TNF-α), monocyte chemoattractant protein-1 (MCP-1), interleukin-6 (IL-6), IL-1β, plasminogen activator inhibitor-1 (PAI-1), resistin, and adipokines, have both vasocrine and paracrine effects on the myocardium [112,113]. Other specific molecules secreted from EAT, such as adiponectin and adipocyte-derived relaxing factors called adipokines, can decrease contraction and vasoconstriction by increasing nitric oxide (NO) release or by reducing reactive oxygen species (ROS) production [114]. In addition, macrophages residing in EAT can release anti-inflammatory cytokines such as IL-10 [115]. EAT may contribute to cardioprotection by the local secretion of anti-inflammatory and anti-atherogenic adipokines such as adiponectin and adrenomedullin [116,117]. Both adiponectin and adrenomedullin are directly secreted from EAT into the coronary circulation, and their mRNA levels are correlated with their intracoronary levels [118,119]. Clinically, both adiponectin and adrenomedullin expression in EAT were significantly reduced in patients with coronary artery disease [118,119]. In a clear demonstration of the paracrine regulation of the cardiac function by PGC1 in mice, we found that chronic iron loading attenuated serum adiponectin concentration, thereby resulting in cardiomyopathy. In addition, adiponectin gene (ADIPOQ) overexpression in the heart after adeno-associated virus delivery (AAV-ADIPOQ) ameliorated cardiac iron deposition and restored the cardiac function in iron-overloaded mice; this occurred via the induced expression of heme oxygenase 1 (HO-1) through the PPARα–PGC-1 complex–dependent pathway in cardiomyocytes [120]. Craige et al., created mice with endothelial-specific loss of function (PGC-1α EC KO) that showed significantly reduced PGC-1α expression as well as decreased endothelial NO synthase (eNOS) expression and NO• bioactivity in response to angiotensin-II-induced hypertension [121]. The authors found that PGC-1α EC KO mice had significantly increased blood pressure with vascular dysfunction compared with Cre control mice. In summary, they showed that endothelial PGC-1α expression is required to exert vascular protection via increased bioactivity of NO• through ERRα-induced expression of eNOS, thus preventing cardiovascular disease.

## 7. Potential for PGC-1α to Modulate Paracrine Regulators in the Heart

PGC-1α is abundantly expressed in tissues with high energy requirements, such as the heart, skeletal muscle, kidney, and BAT [122,123]. In these tissues, PGC-1α controls the expression of genes involved in energy homeostasis, mitochondrial biogenesis, and free FA oxidation function [5,6]. In the heart, cardiomyopathy progression is determined by the amount and the time period of PGC-1α expression. However, the therapeutic window of PGC-1α in cardiomyocytes is relatively narrow because prolonged overexpression of this cofactor leads to uncontrolled mitochondrial proliferation, abnormal sarcomeric structure, and dilated cardiomyopathy [10,124]. Similar phenomena were found in kidney diseases. In the kidney, the basal expression of PGC-1α is stronger in the proximal than the distal tubules, whereas in the glomerulus it is low. A recent study showed aggravated glomerular cell injury when PGC-1 was chronically overexpressed, which is in contrast to the beneficial effects of PGC-1α expression in the proximal tubules promoting acute kidney injury recovery during systemic inflammation [125] or in cisplatin-induced acute renal injury [126]. The cardiac endothelium forms a continuous monolayer of cells that lines the cavity of the heart (endocardial endothelial cells (EECs)) and the luminal surface of the myocardial blood vessels (intramyocardial capillary endothelial cells (IMCEs)). Both EECs and IMCEs can master the contractility of cardiomyocytes by releasing various factors such as NO via endothelial NO-synthase (eNOS), angiotensin II, endothelin-1, peptide growth factors, prostaglandins, and neuregulin-1 (NRG-1) [127]. Craige et al., showed that PGC-1α expression protects the endothelium via increased eNOS expression and NO• bioactivity. ERRα is required for PGC-1α-mediated eNOS expression [121]. Chronic NRG-1 treatment increased oxidative metabolism and mitochondrial activity by enhancing the expression of PGC-1α and PPARδ [128]. Whether PGC-1α can modulate paracrine regulator in the heart needs further investigation. Besides, there are no reports confirming that EAT can act in a paracrine fashion to regulate PGC-1α expression in cardiomyocytes. However, prior reports have indicated that the expression of PGC1α in skeletal muscle may enable the production and release of myokines for the crosstalk between skeletal muscle and other organs. Therefore, future studies should focus on exploring whether PGC-1 in EAT stimulates the secretion of factors that regulate cardiac functions in a paracrine manner, in which cardiac muscle and skeletal muscle can act as endocrine organs.

## 8. PGC-1α Regulates Metabolic Homeostasis in the Liver

The expression of PGC-1α is induced in the liver at birth [129]. Starvation induces PGC-1α expression in the adult liver via glucagon and glucocorticoid (GR) signaling [130]. The fed-to-fasted transition cause metabolic changes in the liver to promote adaptation to nutrient deprivation. These metabolic changes consist in the activation of hepatic gluconeogenesis, FA β-oxidation, heme biosynthesis, bile acid homeostasis, and synthesis and secretion of ketone bodies [131]. In vitro studies in hepatocytes and in vivo studies have shown that PGC-1α is sufficient to activate the hepatic fasting responses, which include gluconeogenesis, ketogenesis, FA β-oxidation, and bile acid homeostasis [130,132,133]. PGC-1α regulates the metabolic adaption to fasting by coactivating key hepatic transcription factors such as HNF4α, PPARα, GR, FOXO1, LXR, and FXR [4]. PGC-1α-KO mice and RNAi-mediated liver-specific PGC-1α-knockdown mice showed defective gluconeogenic gene expression and hepatic glucose production [134,135]. These mice show a tendency for hypoglycemia and hepatic steatosis upon fasting [69]. In addition, PGC-1α stimulates the expression of genes involved in homocysteine metabolism in cultured primary hepatocytes and in the liver [136]. Hepatic PGC-1α protein expression and activation of mitochondrial biogenesis were reduced in a mouse model of hepatic steatosis [137]. PGC-1α plays an important role in exercise-induced hepatic mitochondrial adaptation [138]. PGC-1α expression was lower in the liver of obese, sedentary humans than lean humans [139]. Overexpression of hepatic PGC-1α increased hepatic FA oxidation with decreased TAG storage and secretion in vivo and in vitro [140]. In addition, PGC-1α integrates the mammalian clock and energy metabolism. PGC-1α stimulates the gene expression of the clock genes *Bmal1* (*Arntl*) and *Rev-erbα* (*Nr1d1*) by coactivation of the receptor tyrosine kinase-like orphan receptor family of orphan nuclear receptors. PGC-1α-null mice show abnormal diurnal rhythms of activity, body temperature, and metabolic rate [141]. Therefore, PGC-1α regulates both the fed-to-fasted energy transition and the diurnal rhythm in liver metabolic homeostasis.

## 9. PGC-1α Regulates Kidney Metabolism via Mitochondrial Homeostasis

As a bridge between homeostasis and mitochondrial function, PGC-1α activates NRF-1 and -2, which are nuclear-encoded transcription factors that promote the expression of multiple genes involved in mitochondrial DNA transcription and mitochondrial respiratory chains with anti-oxidative effects [142,143]. In the kidney, PGC-1α is predominantly expressed in the proximal tubules, and enforced expression of PGC-1α in cultured proximal tubular cells increased mitochondrial number, respiratory capacity, and mitochondrial protein level, which indicates the effectiveness of PGC-1α in proximal tubular homeostasis [122,144]. In the septic acute kidney injury (AKI) model, PGC-1α expression in tubular cells was proportionally decreased with an increasing degree of renal impairment. Although mice with PGC-1α gene deletion do not show altered kidney size [69,134], they exhibit increased serum blood urea nitrogen (BUN) and creatinine levels in these models [125], and patients and mouse models with acute and chronic kidney disease commonly show decreased PGC-1α expression accompanied by reduced FA oxidation. In addition, treatment with the PPARγ agonist rosiglitazone could induce PGC-1α expression in the nucleus of renal mesangial cells and significantly ameliorate renal fibrosis in mouse models of diabetic kidney disease. Furthermore, in vitro experiments with cultured renal mesangial cells demonstrated that PGC-1α knockdown increased glucose-induced ROS levels [145]. 

**Figure 2:**
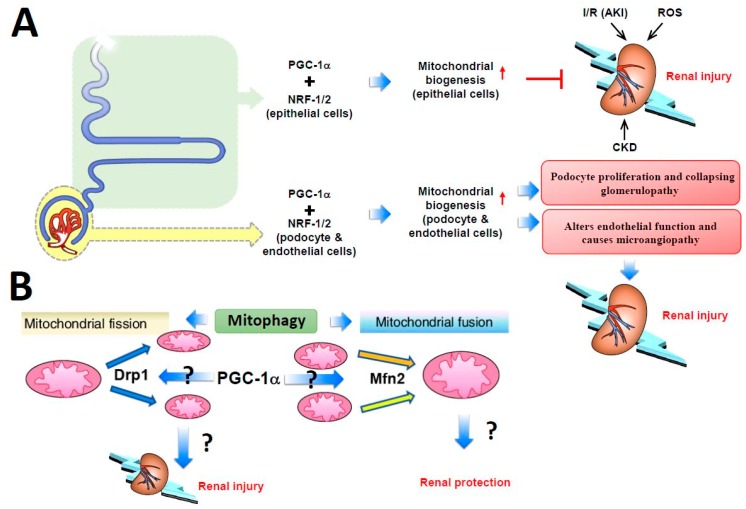
Schematic description of PGC-1α function in renal homeostasis. (**A**) PGC-1α associated with nuclear respiratory factors 1 and 2 (NRF-1/2) has a protective role in renal epithelial cells, including the proximal convoluted tubule, loop of Henle, and distal convoluted tubule, during renal injury by increasing mitochondrial biogenesis in epithelial cells. Bowman’s capsule, podocytes, and endothelial cells have a narrow PGC-1α tolerance. Increased PGC-1α levels in podocytes induce podocyte proliferation and collapsing glomerulopathy development, whereas increased PGC1-α in endothelial cells alters endothelial function and causes microangiopathy, thereby resulting in renal injury. (**B**) The role of mitochondrial fusion and fission in mitophagy. Mitochondrial fusion is promoted by the *Mfn2* gene, whereas *Drp1* promotes mitochondrial fission. Increased *Drp1* and decreased *Mfn2* expression exacerbates tubular damage, thereby contributing to kidney disease; however, studies have shown opposite results and inconsistencies. Whether PGC-1α transcriptionally regulates *Drp1* and *Mfn2* requires further research.

Studies from Rasbach et al., who used tertbutyl hydroperoxide (tBHP), an agent that profoundly depletes cellular glutathione, to induce oxidative stress in the rabbit proximal tubular cell culture system resulted in iron-dependent lipid peroxidation with extensive primary mitochondrial damage [146]. PGC-1α protein level was greatly increased after tBHP treatment, and the increase could be blocked by inhibiting the epidermal growth factor receptor–Src–p38 MAPK axis pathway. However, adenovirus-induced PGC-1α overexpression produced a 25% to 50% increase in mitochondrial number [147], which had a protective effect against tBHP-induced cell damage [144]. Choi et al., found that PGC1-α could attenuate ischemia-reperfusion-induced acute kidney injury by ameliorating the mitochondria dysfunction mediated by p38 signaling [148]. These consistent in vivo and in vitro findings indicate that PGC-1α expression may be increased in the early stage of acute and chronic kidney injury as a compensatory response and PGC-1α can regulate renal tubular mitochondrial biogenesis. Other kidney cells, such as podocytes and endothelial cells, are less metabolically active and have a narrow PGC-1α tolerance. Increasing PGC-1α levels in podocytes induce podocyte proliferation and collapsing glomerulopathy development, whereas increasing PGC-1α levels in endothelial cells alter the endothelial function and cause microangiopathy, thus highlighting the cell type-specific role of PGC-1α in the kidney (Figure 2A).

Defects in mitochondrial fusion–fission homeostasis lead to altered mitochondrial morphology and impaired mitochondrial function and cause tubular damage in acute kidney injury. In addition, the balance between mitochondrial fusion and fission shifts to mitochondrial fission, resulting in mitochondrial fragmentation and then altered mitochondrial structure and renal tubular cell apoptosis [149]. Brooks et al. [149] observed mitochondrial fragmentation and *Drp1* mobilization to the outer mitochondrial membrane in injured tubular cells. *Drp1*, a mitochondrial fission mediator, is activated rapidly after ischemia-reperfusion-induced injury and induces mitochondrial fragmentation and subsequent renal tubular cell apoptosis [149]. By using dominant-negative mutants and RNA interference, Jiang et al., demonstrated that *Drp1* inhibition attenuated mitochondrial fragmentation, preserved mitochondrial integrity, limited renal cell apoptosis, and preserved kidney function. However, pharmacological inhibition or genetic deletion of autophagy-related genes worsened renal injury. These inconsistent results may imply that excessive mitochondrial fission during acute kidney injury is deleterious to organ function, and safe clearance of damaged mitochondria via mitophagy may be protective [150,151]. Meanwhile, primary cultured cells with tissue-specific knockout of *Mfn2*, a mitochondrial fusion mediator, in renal proximal tubular cells were highly sensitive to Bax activation and cytochrome c release, which led to cell apoptosis. However, *Mfn2* is also known to suppress cell proliferative effects via p21Ras, independently of mitochondrial dynamics [152,153]. Such *Mfn2*-mediated hyperplasia suppression may contribute greatly to renal recovery after stress; therefore, reducing *Mfn2* level in proximal tubular cells might actually accelerate organ recovery [154,155]. Gall et al., had shown that conditional knockout of proximal tubule *Mfn2* markedly boosts recovery of renal function and increased rodent survival after acute renal ischemia, partially by activating Ras and ERK1/2 signaling [156]. The above findings indicate, in general, that increased *Drp1* or decreased *Mfn2* levels exacerbate renal tubular damage via an imbalance in mitochondrial fission and fusion, with subsequent enhancement of mitochondrial fragmentation and aggravated acute kidney injury; however, studies with opposite results were also reported (Figure 2B). Further research is needed to investigate whether PGC-1α evokes the performance of the mitochondrial genes via the *Drp1–Mfn2* balance pathway and thus affects the function of the kidney in health or disease.

## 10. PGC-1α Regulates Cancer Metabolism

Metabolic reprogramming occurring in cancer cells refers to the ability to grow and survive under nutrient-starved or stressful microenvironments [157,158]. Increments in glycolysis, glutaminolytic flux, amino acid and lipid metabolism, and mitochondrial biogenesis have been observed in cancer development [159,160,161,162]. Deregulated metabolism is associated with oncogenesis, including the phenomenon of epithelial-to-mesenchymal transition (EMT), a complicated process that enables cancer cells to invade neighboring tissues and migrate to the vasculature [163,164,165]. Among the numerous regulators of cancer metabolism, PGC-1α has been shown to regulate many processes linked to oncogenesis by, for example, promoting the expression of antioxidant genes which protect cells from the detrimental effects of ROS, enhancing the catabolism of glucose and fatty acids, and promoting gluconeogenesis and lipogenesis which perform opposite anabolic functions [166,167,168,169,170]. No specific variant or isoform of PGC-1α has been reported in cancer studies. Some studies have shown that biphasic expression of PGC1α was observed in cancer biopsies or cells of breast cancer [171,172,173,174], melanoma [175,176,177], colon cancer [169,178], and ovarian cancer [179,180,181]. Low PGC-1α levels are associated with a worse outcome in breast and liver carcinomas [171,172,182]. The chemoresistant clear-cell subtype of ovarian carcinoma was identified by the lack of expression of both PGC-1α and mitochondrial transcription factor A (TFAM) [180]. In contrast, some studies showed that the plasma concentrations of PGC1α in breast cancer patients were higher than in healthy groups, and a multivariate analysis showed a correlation between high levels of PGC-1α and worse prognosis [183]. In a report of prostate cancer, androgens signaling via AMPK caused the increment of PGC1α mitobiogenesis, OXPHOS, and glycolysis. Furthermore, findings in mouse xenografts and patient samples suggested that AMPK–PGC1α function was associated with prostate cancer growth [184].

Even though many studies have investigated the role of PGC-1α in cancer by examining its expression via PGC-1α overexpression and siRNA knockdown experiments, the role of PGC-1α in cancer is still controversial. Several studies have shown that PGC-1α has tumor-suppressive effects. PGC-1α overexpression in melanoma cells by adenovirus infection suppressed metastasis via the direct regulation of inhibitor of DNA binding protein 2 (ID2) and the inhibition of transcription factor 4 (TCF4)-mediated gene transcription [177]. Human ovarian cancer cell line Ho-8910 overexpressing PGC-1α has been shown to undergo apoptosis through downregulation of B-cell lymphoma 2 (Bcl-2) and upregulation of Bcl2-associated X protein (Bax) [185]. Wang et al., revealed that increased PGC-1α expression by a PPAR pan-agonist (bezafibrate) upregulated mitochondrial biogenesis, resulting in the inhibition of proliferation and invasion in HeLa, 143B, and MDA-MB-231 cancer cells [186]. Overexpression of PGC-1α by adenovirus infection in HepG2 human hepatoma cells upregulated E-cadherin expression and inhibited cell motility [187]. A study by Torrano et al., showed that PGC-1α inhibited the metastasization of prostate carcinoma via an estrogen-related receptor alpha (ERRα)-dependent transcriptional program [188]. PGC-1α overexpression in HT29 and HCT116 colorectal cancer cells induced apoptosis through ROS accumulation [178]. 

As opposed to the tumor-suppressive role of PGC-1α described above, many reports have shown that PGC-1α is a tumor promoter [169,170,176,184,189,190,191]. Bhalla et al., demonstrated that PGC-1α knockout mice had reduced chemical-induced liver and colon carcinogenesis, suggesting that PGC-1α may induce carcinogenesis [169]. This study reported that PGC-1α stimulates carcinogenesis and tumor growth via the induction of lipogenic enzymes (fatty acid synthase and acetyl-CoA carboxylase) in genetically modified PGC-1α mice [169]. In addition, knockdown PGC-1α significantly induced apoptosis in PGC-1α-positive melanoma cell lines, suggesting that PGC-1α regulates the survival of PGC-1α-positive melanoma cells [176]. PGC-1α promoted prostate cancer cell growth through the activation of androgen receptor [184,189]. It was shown that cell proliferation was inhibited in PGC-1α siRNA knockdown experiments in H1944 lung adenocarcinoma cells [191]. Similarly, overexpression of PGC-1α induced HEK293 cell proliferation and tumorigenesis through the upregulation of Specificity protein 1 (Sp1) and acyl-CoA-binding protein [190]. PGC-1α overexpression or ERRα activation conferred breast cancer cell growth ability, even under hypoxia conditions [170]. Despite the fact that PGC-1α can act as a tumor suppressor and a tumor promoter, there is no explicitly defined mechanism that can explain its dichotomous effects. However, its dual actions can be partially explained by its cell type-specific effects and varied interacting proteins. 

## 11. Conclusions

PPARα and PGC-1α play a central role in metabolic flexibility by driving robust and coordinated changes in the expression of key components of mitochondrial biogenesis and by performing a critical metabolic regulation in many vital organs, including adipose tissue, skeletal muscle, heart, liver, and kidney (Figure 1). Traditionally, in BAT and WAT, mitochondrial biogenesis and BAT gene expression are regulated by PGC-1α. Adrenergic stimulation and reduced temperature trigger signaling cascades including the upregulation of UCP-1 level, thereby resulting in body thermogenesis. In skeletal muscle and WAT, the transcriptional activity of PGC-1α is responsible for the expression of gene networks that control glucose uptake, glycolysis, FA oxidation, the TCA cycle, OXPHOS, and mitochondrial biogenesis and uncoupling. Therefore, increased exercise will increase mitochondrial gene biogenesis and the secretion of myokines (such as irisin), resulting in WAT browning and liver gluconeogenesis and preventing obesity and insulin resistance. In EAT, increased HO-1 expression depends on the PGC-1α–UCP-1 axis, which subsequently decreases free radical and ROS production, thus reducing cardiomyopathy. However, whether long-term PGC-1α overexpression improves or impairs heart or kidney function under disease- or stress-induced remodeling is unclear. In cancer, the dichotomous effects of PGC-1α can be partially explained by its cell type-specific effects and diverse interacting proteins. Therefore, more details in vivo and pre-clinical work are required to assess the usefulness of PGC-1α-inducing drugs in cardiovascular, renal, and cancer therapy.
